# Association of MAPK4 and SOX1-OT gene polymorphisms with cleft lip palate in multiplex families: A genetic study

**DOI:** 10.34172/joddd.2020.021

**Published:** 2020-06-17

**Authors:** Praveen Kumar Neela, Srinivas Reddy Gosla, Akhter Husain, Vasavi Mohan, Sravya Thumoju, Rajeshwari BV

**Affiliations:** ^1^Department of Orthodontics, Kamineni Institute of Dental Sciences, Narketpally. India; ^2^Department of Cranio-maxillofacial Surgery AIIMS, Rishikesh, Hyderabad, India; ^3^Department of Orthodontics, Yenepoya Dental College, Yenepoya University, Mangalore, India; ^4^Department of Genetics and Molecular Medicine, Vasavi Medical and Research Centre, Hyderabad, India; ^5^Surabhi Institute of Medical Sciences, Telangana, India

**Keywords:** Cleft lip palate, Gene, Polymorphism

## Abstract

**Background.** Cleft lip and palate (CLP) is a common congenital anomaly. Many genes, like MAPK4 and SOX-1OT, are associated with its etiology in different populations. High-risk markers on these gene sreported in other populations were not studied in our population. Hence, the study aimed to determine the association of MAPK4 and SOX-1OT polymorphisms in CLP in multiplex families.

**Methods.** Based on inclusion and exclusion criteria, we selected 20 multiplex CLP families for this case‒control study, in which the affected individuals and healthy controls selected from these families were compared. Fifty subjects affected with cleft and 38 unaffected subjects were included in the study. The polymorphisms studied for the association consisted of rs726455 and rs2969972 in the genes SOX-1 OT and MAPK4, respectively. DNA was isolated and sent for genotyping using the MassArray method. Plink, a whole-genome association analysis toolset, was used for statistical analysis.

**Results.** Both polymorphisms followed Hardy–Weinberg equilibrium. The rs726455 of SOX-1OT yielded a P-value of 0.983 and an allelic odds ratio (OR) of 0.983. For rs2969972 of MAPK4, the P-value was 0.04 (significant), and the allelic OR was 0.51. Minor allele frequency (MAF) in the unaffected subjects was more than the MAF in the affected subjects for rs2969972.

**Conclusion.** The results suggested that polymorphism rs726455 on SOX-1OT was not associated with familial cases of CLP. Since MAF in the unaffected subjects was more than the MAF-affected subjects, rs2969972 on MAPK4 is protective in the multiplex families.

## Introduction

Cleft lip and palate (CLP) is one of the most common congenital deformities occurring in humans. The affected individuals might have a cleft of the lip or palate or both. Cleft lip and palate is more common in males compared to an isolated cleft palate in females, but their prevalence varies according to ethnicity and geographical location.^1^ According to Reddy et al,^2^ the incidence of clefts in India is around1:800 to 1:1000, and three infants are born with some type of cleft every hour.Worldwide surveys have shown that the frequency of CLP varies significantly from one country to another. It is the lowest in Africans (1:2500), and North American Indians and East Asians have the highest prevalence (1:500).^1^ 70% of the CLP cases are non-syndromic and occur as isolated cases. In contrast, 30% of clefts are syndromic and are associated with a few other deformities.^3,4^

The etiology of (CLP) is very complicated because of the relevant congenital anomalies.^5^ The etiology is polygenic and multifactorial, involving both genetic and environmental factors,^6^ including heredity, consanguinity, fetal environment, demographic factors, other factors like drugs, vitamins, alcohol consumption and smoking during pregnancy, infections, diet, etc.^7^ Among all these etiologies, consanguinity is an important factor. Neela et al^8^ reported in a 13-year retrospective study that20.02%ofcleftpatients had consanguineous parents.Ram Kumar Sah and Rajesh Powar^9^ reported that consanguineous marriage was noted in 48.9% of the parents.

The lips form during the 4th-7th weeks of fetal life, whereas the palate is formed between the sixth and ninth weeks. Cleft lip occurs when the lateral nasal and maxillary processes forming the craniofacial complex do not fuse completely.^10^ Many genes play an essential role in the formation of lips and palate based on the stages during growth and development.^11^ Genes like HOX, SSH, MSX, CDH1, LHX, DHFR, BMP4, GSC, FGF1, DLX, PRR, etc.,are responsible for the migration and proliferation of neural crest cells in the lips and palatal area.^11^

Several genetic studies on CLP have been conducted on many high-risk polymorphisms in different populations on isolated and familial cases of CLP. Some of the genes on which single or multiple polymorphisms have been studied include FOXE1, GLI2, MSX2, SKI, SATB2, SPRY2, TGFB3, TGFA, P63, RUNX2, BMP4, PAX7, TGFB3, GRHL3, IRF6, NAT2, SDC2, BCL3, PVRL1, etc.^12-19^

MAPK4 (mitogen-activated protein kinase 4) is a member of the mitogen-activated protein kinase family. Tyrosine kinase growth factor receptors activate mitogen-activated protein kinases, which then translocate into the nucleus and phosphorylate nuclear targets. Alternative splicing results in multiple transcript variants.^20^ SOX1-OT (SOX1 overlapping transcript) is involved in early embryogenesis and maintenance of neural stem cells.^21^

Presently, the literature review reveals no study on the association between the MAPK4 & SOX1-OTgene polymorphisms and the risk of developing non-syndromic cleft lip and/or palate NSCL/P in the Indian population. Furthermore, previous studies have evaluated one or two families. Polymorphisms must be analyzed for their etiology in several multiplex families. Since the two genes mentioned above are essential in the embryogenesis, the current study aimed to assess the possible connection between polymorphisms rs726455 (rs- reference SNP) and rs2969972 of MAPK4 and SOX1-OT genes, respectively, in several multiplex families in the Indian population.

## Methods

The present study was performed following the Helsinki Declaration. GSR Institute of Craniofacial surgery was selected as it is a high-volume center where patients from all over the country refer to for treatment. Twenty multiplex families of NSCLP were selected after excluding the syndromic cases, patients with associated anomalies, and mental retardation. A multiplex family is a family in which a person diagnosed with a complex genetic disorder has a first- or second-degree relative with the same disorder. All the cleft subjects were familial non-syndromic cases with any type of phenotype. A total of 88 subjects, including 50 NSCLP (non-syndromic cleft lip and/or palate) patients and 38 healthy subjects were included from these 20 families. All the affected subjects were from different multiplex families. After taking consent from all the participants, 4‒5 mL of venous blood was taken in EDTA tubes. These tubes were stored in ice packs and kept in a Thermocol box to maintain temperature during transport, until they are refrigerated at 4ºC. Genomic DNA was isolated using the salting-out method.^22^ An ultraviolet spectrometer was used to calculate the average 260/280 nm ratio to assess the purity and concentration of DNA.The ratio of absorbance readings at the two wavelengths should be between 1.8 and 2.0 (i.e., A260/A280 = 1.7‒2.0). Later, the DNA was sent for SNP genotyping of the polymorphisms (Table 1).

**Table 1:** List of SNPs and the related genes

**Polymorphism**	**Gene**	**Normal Sequence**	**Ancestral Allele**
rs726455	SOX1-OT	C/T-FWD	C
rs2969972	MAPK4	A/G-FWD	G

Agena Bio MassARRAY (Agena Bioscience, Inc., San Diego, CA, USA) platform using iPLEX Gold technology was used for the SNP genotyping. This system is a non-fluorescent, highly accurate detection platform utilizing Matrix-Assisted Laser Desorption/Ionization - Time of Flight (MALDI-TOF) mass spectrometry.^23^ The assay was designed using proprietary Agena software (Assay Design Suite 2.0). The assay design was used to design primers. MassArray workflow was followed according to its protocol, and finally, the samples were run through the analyzer. Agena’s SpectroTyper 4.0 software (San Diego, CA, USA) was used, which automatically generates information that helps in the identification of alleles (homozygous or heterozygous). The data obtained from the analyzer software were sent for statistical analysis.

### Statistical analysis

The SNP allele data of the probands and controls derived from the MassArray system were subjected to statistical analysis. PLINK software (Version 1.09) was used for this study.^24^ It is a free and open-source whole-genome association toolset designed to perform a variety of analyses ranging from basic to large-scale in a computationally effective means. Using the same PLINK, genotype distribution was used to calculate the Hardy–Weinberg equilibrium (HWE). Statistical comparisons between the affected and unaffected subjects were carried out using PLINK software. Odds ratios (OR) and 95% confidence intervals were provided. The allelic association was tested using chi-squared test. For nominal association, the statistical significance level was set to α=0.05

## Results

We observed that both polymorphisms (SNPs) on genes SOX1-OT and MAPK4 were in HWE. Genotypic and allelic frequencies of polymorphisms on SOX1-OT and MAPK4 analyzed are shown in Table 2.

**Table 2:** Allelic Association for SNPs analyzed

**SNP**	**A1**	**F_A**	**F_U**	**A2**	**CHISQ**	**P**	**OR (95% CI)**
**rs726455**	T	0.38	0.3816	C	0.0004566	0.983	0.9933
**rs2969972**	A	0.25	0.3947	G	4.211	0.04017	0.5111

†Chi-squared test. SNP = Single Nucleotide Polymorphism, A1 = Major Allele (Wild Allele), ††F_A = Minor Allele Frequency Affected, F_ U = Minor allele Frequency Unaffected, A2-Minor Allele (Mutant), CHISQ = Chi-squared, P = P-value, OR = Odds Ratio, CI = Confidence Interval. The P-values <0.05 is significant.

The polymorphism rs726455 on SOX1-OT gene showed no significance in association analysis (P=0.983, OR=0.9933). However, the polymorphism rs2969972 on MAPK4 gene exhibited a P-value of 0.04017 and an odds ratio of 0.5111. This indicates that polymorphism rs2969972 significantly decreased the risk of NSCLP as the minor allele frequency (MAF) in the unaffected subjects was more than the minor allele frequency in the affected patients.

## Discussion

Genetic and environmental factors play a crucial role in the development of CLP. Wide genomic variations are considered in the etiology of both syndromic and non-syndromic variants of CLP. Mutations in several regions of the genes were reported, including some transcription factors (IRF6, MSX1, TBX22), growth factors (TGFA, TGFb3), metabolism genes (CYP1A1, GSTM1, NAT2), and some genes involved in immune response (PVRL1).^25,26^ However, there are conflicting reports on the involvement of these genes in various populations.Moreover, recent genome-wide association studies (GWAS) have identified different chromosomal loci that might harbor common variants associated with increased risk of CLP, including 1p22, 1p36, 2p21, 3p11.1, 8q21.3, 8q24, 9q22, 10q25, 15q22, 17p13, 17q22, and 20q12.^27^ The SOX1-OT and MAPK4 genes are involved in early embryogenesis and protein binding, which is very important in midface development and upper lip fusion. Genetic variations in our human genome in the form of SNPs are abundant and responsible for differences in the phenotype in and among different populations.

In the present study, we studied the possibility of an association between polymorphisms rs726455 on gene SOX1-OT and rs2969972 on gene MAPK4 and their role in NSCLP in familial cases. The results showed no significance in association analysis for polymorphism rs726455 on SOX1-OT. However, the polymorphism rs2969972 on MAPK4 was protective despite its significance. Contrary to the general findings, our results revealed that rs2969972 polymorphism significantly decreased the risk of NSCLP as the MAF in the unaffected was more than that in the affected.

In a study by Radhakrishna et al^28^ on two pedigrees with non-syndromic cleft lip palate, the high-risk marker rs726455 showed no significance.^28^ However, in the same region on 13q33.1-34 marker, rs1830756 showed a significant association with NSCLP. A research by Beiraghi et al,^29^ using genome-wide linkage analysis, showed proof of linkage for the marker rs728683 on 18q21.1 region.^29^ However, in the same region, rs2969972 on gene MAPK4 showed no significance for CLP and was protective for normal subjects.

A Fine-Mapping study on 18q21.1 locus showed that MYO5B SNP rs183559995 had an odds ratio of 18.09.^30^ Other SNPs also exhibited significant association with NSCL/P risk: rs1450425 (LOXHD1), rs6507992 (SKA1), rs78950893 (SMAD7), rs8097060, rs17713847 (SCARNA17), rs6507872 (CTIF), rs8091995 (CTIF), and rs17715416 (MYO5B). However, in the present study, rs2969972 polymorphism, present in the same locus, significantly decreased the risk of NSCLP as the MAF in the unaffected was more than that in the affected. A literature search showed a varied disparity in the association of various markers in various populations. The marker identified as a risk in one particular population might not be identified again in the same population or different populations as a risk factor. The differences in the results might be attributed to ethnic variations, environmental differences, and the complexity of the genetic etiology of NSCLP.

## Conclusion

MAPK4 and SOX1-OTpolymorphisms analyzed in the present study were not associated with an increased risk of non-syndromic cleft lip palate. Our results revealed that rs2969972 polymorphism significantly decreased the risk of CLP in unaffected subjects as the MAF in the unaffected was higher than that in affected individuals. Hence, it is protective, or it decreased the CLP risk in the analyzed subjects in multiplex families of CLP.

## Authors’ Contributions

PKN was responsible for the concept, design, the definition of intellectual content, literature search, experimental data acquisition, data analysis, manuscript preparation, manuscript editing, and manuscript review of the study. SRG was responsible for the concept, design, the definition of intellectual content, literature search, data acquisition, data analysis, manuscript preparation, manuscript editing, and manuscript review. AH was responsible for the concept, design, literature search, data acquisition, data analysis, manuscript preparation, manuscript editing, and manuscript review. VM was responsible for the concept, design, literature search, data acquisition, data analysis, manuscript editing, and manuscript review. ST was responsible for the design, manuscript preparation, data acquisition, data analysis, manuscript editing, and manuscript review. RBV was responsible for the design, manuscript preparation, data acquisition, data analysis, manuscript editing, and manuscript review. All the authors have read and approved the final manuscript.

## Acknowledgments

We thank the subjects for their participation in this study. We are also grateful to Dr. D.V.S. Sudhakar PhD, Post-Doctoral Fellow at the Centre for Cellular and Molecular Biology, Hyderabad, India, for his assistance in the analysis of raw genomic data.

## Funding

Not Applicable.

## Competing interests

The authors declare no competing interests with regards to the authorship and/or publication of this article.

## Ethics Approval

The ethics approval for the study was given by the Independent Ethics Committee of the GSR Institute of Craniofacial and Facial Plastic Surgery. Consent was obtained from the subjects who participated in the research to publish the data.
